# Fast universal quantum gates on microwave photons with all-resonance operations in circuit QED

**DOI:** 10.1038/srep09274

**Published:** 2015-03-19

**Authors:** Ming Hua, Ming-Jie Tao, Fu-Guo Deng

**Affiliations:** 1 Department of Physics, Applied Optics Beijing Area Major Laboratory, Beijing Normal University, Beijing 100875, China

## Abstract

Stark shift on a superconducting qubit in circuit quantum electrodynamics (QED) has been used to construct universal quantum entangling gates on superconducting resonators in previous works. It is a second-order coupling effect between the resonator and the qubit in the dispersive regime, which leads to a slow state-selective rotation on the qubit. Here, we present two proposals to construct the fast universal quantum gates on superconducting resonators in a microwave-photon quantum processor composed of multiple superconducting resonators coupled to a superconducting transmon qutrit, that is, the controlled-phase (c-phase) gate on two microwave-photon resonators and the controlled-controlled phase (cc-phase) gates on three resonators, resorting to quantum resonance operations, without any drive field. Compared with previous works, our universal quantum gates have the higher fidelities and shorter operation times in theory. The numerical simulation shows that the fidelity of our c-phase gate is 99.57% within about 38.1 ns and that of our cc-phase gate is 99.25% within about 73.3 ns.

Quantum computation and quantum information processing have attached much attention^1^ in recent years. A quantum computer can factor an *n*-bit integer exponentially faster than the best known classical algorithms and it can run the famous quantum search algorithm, sometimes known as Grover's algorithm, which enables this search method to be speed up substantially, requiring only 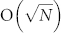 operations, faster than the classical one which requires O(*N*) operations^1^. Universal quantum gates are the key elements in a universal quantum computer, especially the controlled-phase (c-phase) gate or its equivalent gate – the controlled-not (CNOT) gate. C-phase gates (or CNOT gates) assisted by single-qubit rotations can construct a universal quantum computing. Compared to the synthesis with universal two-qubit entangling gates and single-qubit gates, the direct implementation of a universal three-qubit quantum gate [controlled-controlled-phase (cc-phase) or controlled-controlled-not (Toffoli) gate] is more economic and simpler as it requires at least six CNOT gates^2^ to synthesize a Toffoli gate which is equivalent to a cc-phase gate. By far, there are some interesting physical systems used for the construction of universal quantum gates, such as photons^3^,^4^,^5^,^6^,^7^,^8^,^9^, nuclear magnetic resonance^10^,^11^,^12^,^13^,^14^, quantum dots^15^,^16^,^17^,^18^,^19^,^20^,^21^,^22^, diamond nitrogen-vacancy center^23^,^24^,^25^, and cavity quantum electrodynamics (QED)^26^,^27^.

Circuit QED, composed of superconducting Josephson junctions (act as the artificial atoms) and a superconducting resonator (acts as a cavity and quantum bus)^28^,^29^,^30^,^31^,^32^,^33^,^34^, is a promising implementation of cavity QED and it has the excellent features of the good scalability and the long coherence time. It has been used to realize the strong and even ultra-strong coupling between a resonator and a superconducting qubit^28^,^35^, and complete some basic tasks of the quantum computation on the superconducting qubits. For example, DiCarlo *et al.*^36^ demonstrated the c-phase gate on two transmon qubits assisted by circuit QED in 2009. In 2014, Chow *et al.*^37^ experimentally implemented a strand of a scalable fault-tolerant quantum computing fabric. In 2012, Fedorov *et al.*^38^ implementated a Toffoli gate and Reed *et al.*^39^ realized the three-qubit quantum error correction with superconducting circuits. In 2014, Barends *et al.*^40^ realized the c-phase gate on every two adjacent Xmon qubits with a high fidelity in a five-Xmon-qubit system assisted by circuit QED. DiCarlo *et al.*^41^ prepared and measured the three-qubit entanglement in circuit QED in 2010, and Steffen *et al.*^42^ realized the full deterministic quantum teleportation with feed-forward in a chip-based superconducting circuit architecture in 2013.

In a high-quality resonator, a microwave photon always has the longer life time than that of a superconducting qubit^43^, which makes the resonator a good candidate for quantum information processing based on the basis of Fock states. With a superconducting qubit coupled to a resonator, Hofheinz *et al.*^44^ realized the generation of a Fock state in 2008. In the same year, Wang *et al.*^45^ realized the measurement of the decay of Fock States. Hofheinz *et al.*^46^ demonstrated the synthesis of an arbitrary superposition of Fock states in 2009. With two qubits coupled to three resonators, Merkel and Wilhelm^47^ proposed a scheme for the generation of the entangled NOON state on two resonator qudits (with *d* levels) in 2010. In 2011, Wang *et al.*^48^ demonstrated in experiment the generation of the entangled NOON state on two resonators. With a qubit coupled to two resonators, Johnson *et al.*^49^ realized the single microwave-photon non-demolition detection in 2010 and Strauch^50^ exploited the all-resonant method to control the quantum state of superconducting resonators and gave some theoretic schemes for Fock state synthesis, qudit logic operations, and synthesis of NOON states in 2012. With a qubit coupled to multiple resonators, Yang *et al.* proposed a theoretic scheme for the generation of the entangled Greenberger-Horne-Zeilinger state on resonators based on the Fock states^51^ in 2012 and entangled coherent states of four microwave resonators^52^ in 2013.

Besides the entanglement generation for quantum information processing, resonator qudits can also be used for quantum computation, that is, universal quantum logic gates^53^,^54^,^55^. In 2011, Strauch^53^ gave an interesting scheme to construct the quantum entangling gates on the two resonator qudits based on the arbitrary Fock states, by using the two-order coupling effect of the number-state-dependent interaction between a superconducting qubit and a resonator in the dispersive regime, discovered by Schuster *et al.*^56^ in 2007. The operation time of his c-phase gate on two resonator qudits with the basis of the Fock states |0〉*_r_* and |1〉*_r_* is 150 ns. In a processor with one two-energy-level charge qubit coupled to multiple resonators, Wu *et al.*^54^ presented an effective scheme to construct the c-phase gate on two resonators with the number-state-dependent interaction between the qubit and two resonators in 2012. Its operation time is 125 ns. In a similar processor, we gave a scheme for the construction of the c-phase gate (cc-phase gate) on two (three) resonator qubits^55^ (only working under the subspace of Fock states {|0〉*_r_*, |1〉*_r_*}), by combining the number-state-dependent selective rotation between a superconducting transmon qutrit (just the three lowest energy levels are considered) and a resonator (two resonators) and the resonance operation on the rest resonator and the qutrit in 2014. The fidelity of our c-phase (cc-phase) gate can reach 99.5% (92.9%) within 93 (124.6) ns in theory, without considering the decoherence and the dephasing rates of the qutrit and the decay rate of the microwave-photon resonators.

In this paper, we exploit the all-resonance-based quantum operations on a qutrit and resonators to design two schemes for the construction of the c-phase and the cc-phase gates on resonators in a processor composed of multiple microwave-photon resonators coupled to a transmon qutrit far different from the previous works for the c-phase and cc-phase gates on resonators based on the second-order couplings between the qubit and the resonators^53^,^54^,^55^. Our simulation shows that the fidelity of our c-phase gate on two microwave-photon resonators approaches 99.57% within the operation time of about 38.1 ns and that of our cc-phase gate on three resonators is 99.25% within about 73.3 ns. Our all-resonance-based universal quantum gates on microwave-photon resonators without classical drive field are much faster than those in similar previous works^53^,^54^,^55^.

## Results

### All-resonance-based c-phase gate on two resonator qubits

**Figure 1:**
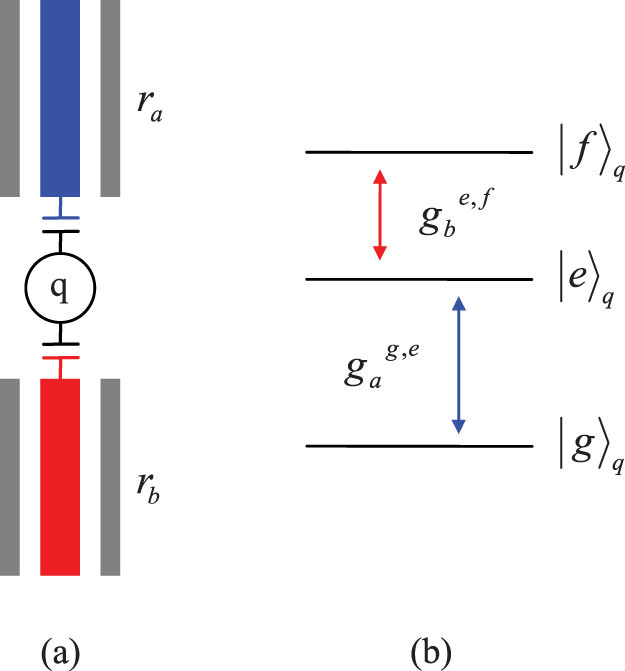
(a) The schematic diagram for our c-phase gate on two microwave-photon resontors in circuit QED. It contains two high-quality superconducting resonators (*r_a_* and *r_b_*) capacitively coupled to a superconducting quantum interferometer device (SQUID) which acts as a superconducting transmon qutrit (*q*), whose transition frequency can be tuned by the external magnetic flux. (b) The schematic diagram for the three lowest energy levels of the qutrit with small anharmonicity. 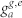 is the coupling strength between *r_a_* and the qutrit with the transition |*g*〉*_q_* ↔ |*e*〉*_q_*. 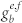 is the coupling strength between *r_b_* and the qutrit with the transition |*e*〉*_q_* ↔ |*f*〉*_q_*.

Let us consider the quantum system composed of two high-quality superconducting resonators coupled to a transmon qutrit with the three lowest energy levels, i.e., the ground state |*g*〉*_q_*, the excited state |*e*〉*_q_*, and the second excited state |*f*〉*_q_*, shown in Fig. 1. In the interaction picture, the Hamiltonian of the system is (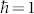):
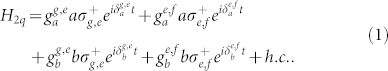
Here, 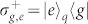 and 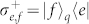 are the creation operators for the transitions of the qutrit |*g*〉*_q_* → |*e*〉*_q_* and |*e*〉*_q_* → |*f*〉*_q_*, respectively. *a*^+^ and *b*^+^ are the creation operators of the resonators *r_a_* and *r_b_* (labeled as *a* and *b* in subscripts), respectively. 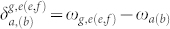 and *ω_g_*_,*e*(*e*,*f*)_ = *E_e_*_(*f*)_ − *E_g_*_(*e*)_. *E_i_* is the energy for the level |*i*〉*_q_* of the qutrit. *ω_a_* and *ω_b_* are the transition frequencies of the resonators *a* and *b*, respectively. 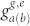 and 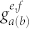 are the coupling strengths between the resonator *r_a_* (*r_b_*) and the qutrit *q* with these two transitions. Tuning the transition frequencies of the transmon qutrit and the coupling strength between the transmon qutrit and each resonator^57^,^58^,^59^, one can turn on and off the interaction between the qutrit and each resonator effectively^60^.

**Figure 2:**
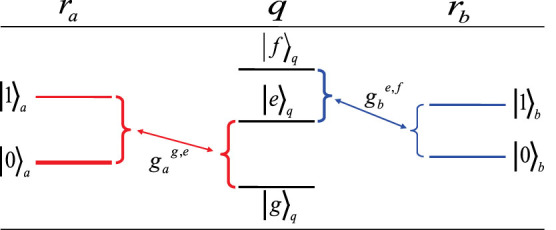
The principle and the steps of our c-phase gate on *r_a_* and *r_b_* with all-resonance operations.

Let us suppose that the general initial state of the system is
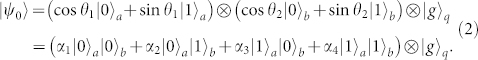
Here, *α*_1_ = cos*θ*_1_cos*θ*_2_, *α*_2_ = cos*θ*_1_sin*θ*_2_, *α*_3_ = sin*θ*_1_cos*θ*_2_, and *α*_4_ = sin*θ*_1_sin*θ*_2_. The all-resonance-based c-phase gate on two microwave-photon resonators can be constructed with three steps, shown in Fig. 2. We describe them in detail as follows.

Step i), by tuning off the interaction between the transmon qutrit and *r_b_*, and resonating *r_a_* and the two lowest energy levels |*g*〉*_q_* and |*e*〉*_q_* of the qutrit (*ω_a_* = *ω_g_*_,*e*_) with the operation time of 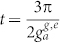, the state of the system can be evolved into
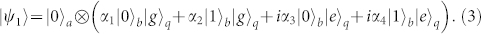


Step ii), by turning off the interaction between the qutrit and *r_a_*, and resonating *r_b_* and the two energy levels |*e*〉*_q_* and |*f*〉*_q_* of the qutrit (*ω_b_* = *ω_e_*_,*f*_) with the operation time of 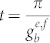, the state of the system can evolve from |*ψ*_1_〉 into
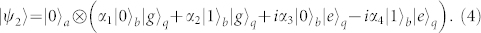


Step iii), by turning off the interaction between the qutrit and *r_b_*, and resonating *r_a_* and the energy levels |*g*〉*_q_* and |*e*〉*_q_* of the qutrit with the operation time of 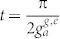, one can get the final state of the system as
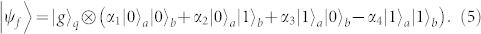
This is just the c-phase gate operation on the resonators *r_a_* and *r_b_*, which indicates that a *π* phase shift takes place only if there is one microwave photon in each resonator.

If the operation time in step i) is taken to 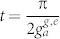, one can get the final state of the system as
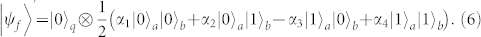
This is just the result of another c-phase gate operation on the resonators *r_a_* and *r_b_*, which indicates that the *π* phase shift happens only if there is one microwave photon in *r_a_* and no microwave photon in *r_b_*.

To show the feasibility of the resonance processes for constructing our c-phase gate, we numerically simulate its fidelity and operation time with the feasible experiential parameters. The evolution of the system composed of these two resonators and the transmon qutrit can be described by the master equation^28^,^61^
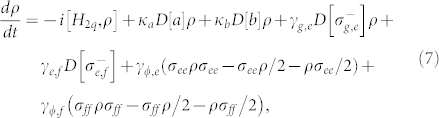
where *D*[*L*]*ρ* = (2*LρL*^+^ − *L*^+^*Lρ* − *ρL*^+^*L*)/2 with 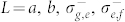. *σ_ee_* = |*e*〉*_q_*〈*e*| and *σ_ff_* = |*f*〉*_q_*〈*f*|. *κ_a_* (*κ_b_*) is the decay rate of the resonator *r_a_* (*r_b_*), *γ_g_*_,*e*_ (*γ_e_*_,*f*_) is the energy relaxation rate of the qutrit with the transition |*e*〉 → |*g*〉 (|*f*〉 − |*e*〉), and *γ_ϕ_*_,*e*_ and *γ_ϕ_*_,*f*_ are the dephasing rates of the levels |*e*〉 and |*f*〉 of the qutrit, respectively. For simplicity, the parameters for our numerical simulation are chosen as: 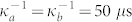, 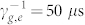, 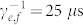, 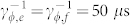, *ω_a_*/(2*π*) = 5.5 GHz, and *ω_b_*/(2*π*) = 7.0 GHz. In the first step, we chose *ω_g_*_,*e*_/(2*π*) = 5.5 GHz, *ω_e_*_,*f*_/(2*π*) = 4.7 GHz, 
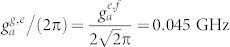
, and 
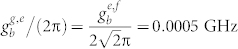
. In the second step, *ω_g_*_,*e*_/(2*π*) = 7.8 GHz, *ω_e_*_,*f*_/(2*π*) = 7.0 GHz, 
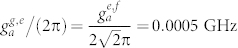
, and 
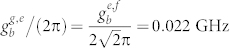
. The parameters in the third step are the same as those in the first step. It worth noticing that the long coherence time of the transmon qubit with 50 μs, the high quality factor of a 1D superconducting resonator with above 10^6^, and the tunable coupling strength of a charge qubit and a resonator with from 200 KHz to 43 MHz have been realized in experiments^59^,^62^,^63^. For superconducting qutrits, the typical transition frequency between two neighboring levels is from 1 GHz to 20 GHz^64^,^65^.

**Figure 3:**
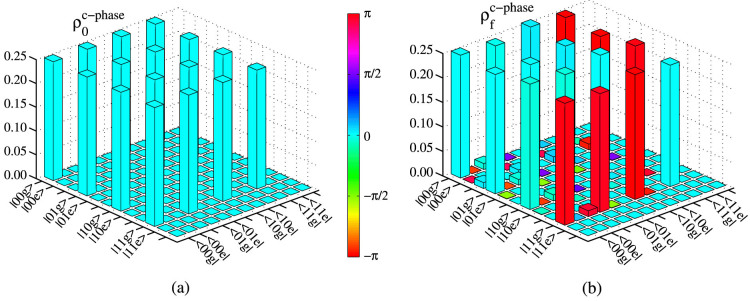
(a) The density operator *ρ*_0_ of the initial state |*ψ*_0_〉 of the quantum system composed of the two resonator qudits and the superconducting qutrit for constructing our c-phase gate. (b) The density operator 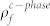. Here we take 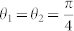.

The fidelity of our c-phase gate is defined as
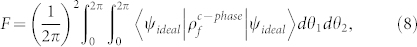
where |*ψ_ideal_*〉 is the final state |*ψ_f_*〉 of the system composed of the resonator qubits *r_a_* and *r_b_* after an ideal c-phase gate operation is performed with the initial state |*ψ*_0_〉, which is obtained by not taking the dissipation and dephasing into account. 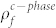 is the realistic density operator after our c-phase gate operation on the initial state |*ψ*_0_〉. Our simulation shows that the fidelity of our c-phase gate is 99.57% within the operation time of about 38.1 ns. Taking 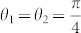 as an example, the density operators of the initial state and the final state are shown in Fig. 3 (a) and (b), respectively.

In fact, by using the resonance operations, one can also construct the swap gate on two resonator qubits simply with our device by the five steps shown in Tab. 1.

### Cc-phase gate on superconducting resonators

Our cc-phase gate is used to perform a minus phase manipulation on the three resonator qubits only if the resonators *r_a_*, *r_b_*, and *r_c_* are in the state |1〉*_a_*|1〉*_b_*|0〉*_c_*.

**Figure 4:**
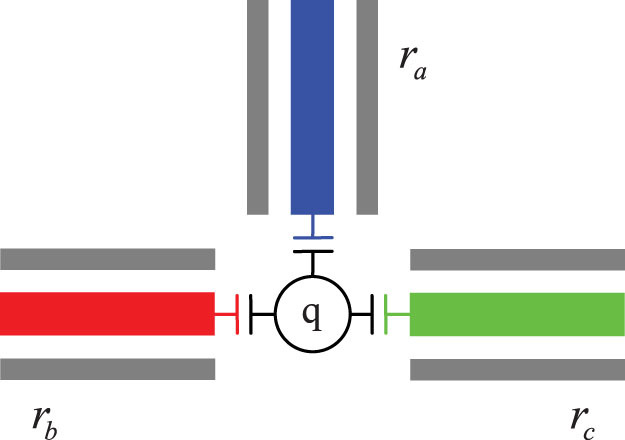
The schematic diagram for our cc-phase gate on three microwave-photon resonators with all-resonance operations in circuit QED. *r_a_*, *r_b_*, and *r_c_* are three high-quality resonators and they are capacitively coupled to the transmon qutrit *q*.

Our device for the cc-phase gate on the three high-quality superconducting resonators *r_a_*, *r_b_*, and *r_c_* which are coupled to the transmon qutrit *q* is shown in Fig. 4. In the interaction picture, the Hamiltonian of the whole system composed of the three resonators and the qutrit is:
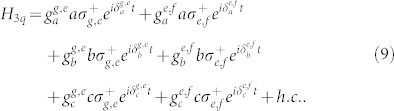
Suppose that the initial state of the system is
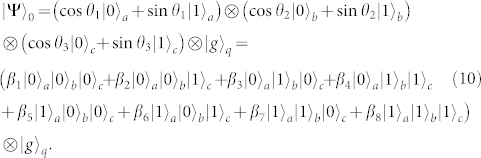
Here, *β*_1_ = cos*θ*_1_cos*θ*_2_cos*θ*_3_, *β*_2_ = cos*θ*_1_cos*θ*_2_sin*θ*_3_, *β*_3_ = cos*θ*_1_sin*θ*_2_cos*θ*_3_, *β*_4_ = cos*θ*_1_sin*θ*_2_sin*θ*_3_, *β*_5_ = sin*θ*_1_cos*θ*_2_cos*θ*_3_, *β*_6_ = sin*θ*_1_cos*θ*_2_sin*θ*_3_, *β*_7_ = sin*θ*_1_sin*θ*_2_cos*θ*_3_, and *β*_8_ = sin*θ*_1_sin*θ*_2_sin*θ*_3_. The cc-phase gate on three resonator qubits can be constructed with nine resonance operations between the qutrit and the resonators. The detailed steps are described as follows.

First, turning off the interaction between *q* and *r_b_* and that between *q* and *r_c_*, and resonating *r_a_* and *q* with the transition |*g*〉*_q_* ↔ |*e*〉*_q_* (*ω_a_* = *ω_g_*_,*e*_), the state of the whole system becomes
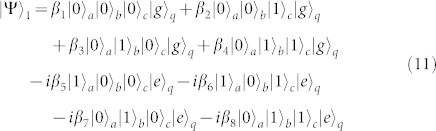
after the interaction time of 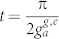.

Second, turning off the interaction between *q* and *r_a_* and that between *q* and *r_c_*, and tuning the frequency of *r_b_* or *q* to make *ω_b_* = *ω_e_*_,*f*_, one can complete the resonance manipulation on *r_b_* and *q* with the transition |*e*〉*_q_* ↔ |*f*〉*_q_*. The state of the whole system can be changed into
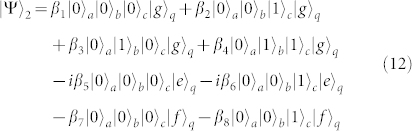
after the operation time of 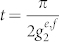.

Third, repeating the same operation as the one in the first step, the state of the whole system can be evolved into
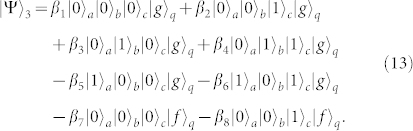


Fourth, turning off the interaction between *q* and *r_b_* and that between *q* and *r_c_*, and resonating *r_a_* and *q* with the transition |*e*〉*_q_* ↔ |*f*〉*_q_*, the state of the whole system evolves from |Ψ〉_3_ into
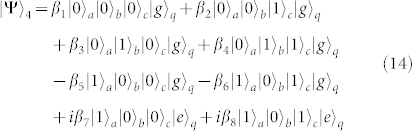
after the operation time of 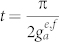.

Fifth, turning off the interaction between *q* and *r_a_* and that between *q* and *r_b_*, and resonating *r_c_* and *q* with the transition |*e*〉*_q_* ↔ |*f*〉*_q_* (*ω_c_* = *ω_e_*_,*f*_), the state becomes
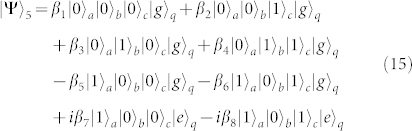
after the operation time of 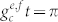.

Sixth, repeating the fourth step, the state of the whole system becomes
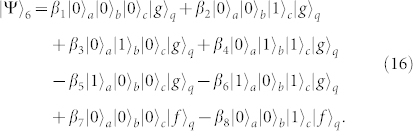


Seventh, taking the same manipulation as the one in the first step, the system is in the state
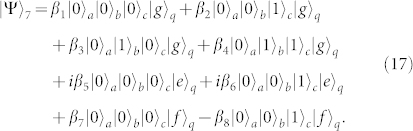


Eighth, repeating the second step, the state of the system evolves from |Ψ〉_7_ into
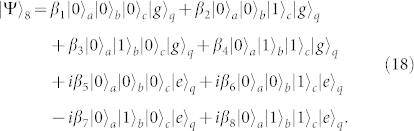


Ninth, repeating the first step, one can get the final state of the whole system as follows
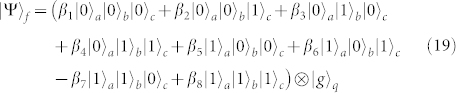
This is just the result of our cc-phase gate on the three microwave-photon resonators.

The evolution of the system composed of three resonators coupled to the transmon qutrit can be described by the master equation
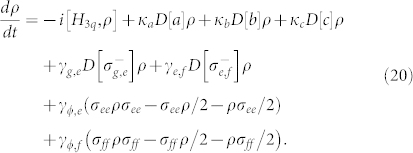
Here *κ_c_* is the decay rate of the resonator *r_c_*. In our simulation for the fidelity of our cc-phase gate, the parameters of the system are chosen as: *ω_a_*/(2*π*) = 5.5 GHz, *ω_b_*/(2*π*) = 7.0 GHz, *ω_c_*/(2*π*) = 8.0 GHz, 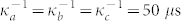, and *ω_g_*_,*e*_/(2*π*) − *ω_e_*_,*f*_/(2*π*) = 800 MHz. The energy relaxation rates and the dephasing rates of the transmon qutrit are chosen the same as those in the construction of our c-phase gate. The details for the parameters chosen in each step for the simulation of our cc-phase gate are shown in Table 2.

Let us define the fidelity of our cc-phase gate as
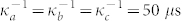
where |*ψ_ideal_*〉 is the final state |Ψ〉*_f_* of the system composed of three resonator qubits *r_a_*, *r_b_*, and *r_c_* after an ideal cc-phase gate operation when the initial state of the system is |Ψ〉_0_, without considering the dissipation and the dephasing. 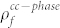 is the realistic density operator after our cc-phase gate operation on the initial state |Ψ〉_0_. We numerically simulate the fidelity of our cc-phase gate, by taking the dissipation and the dephasing into account. The fidelity of our cc-phase gate is 99.25% within the operation time of about 73.3 ns.

**Figure 5:**
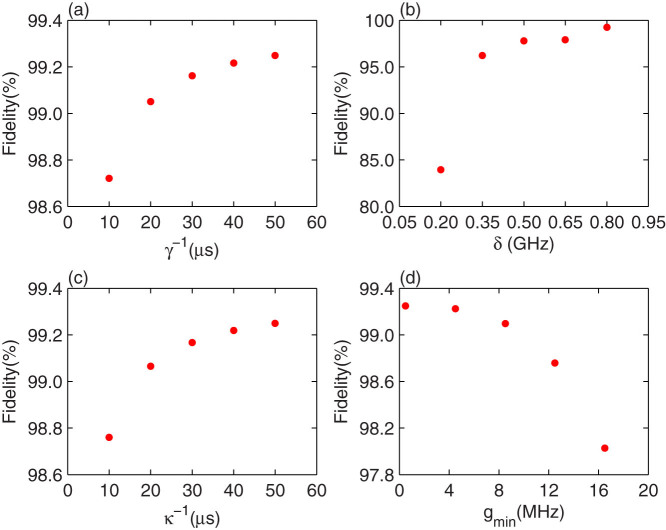
The fidelity of the cc-phase gate varying with the parameters: (a) the energy relaxation rate of the qutrit *γ*, (b) the anharmonicity *δ* = *ω_g_*_,*e*_ − *ω_e_*_,*f*_, (c) the decay rate of resonators with *κ_a_* = *κ_b_* = *κ_c_* ≡ *κ*, and (d) the minimum value of tunable coupling strength *g_min_*. Here 2*γ_e_*_,*f*_ = *γ_g_*_,*e*_ = *γ_ϕ_*_,*e*_ = *γ_ϕ_*_,*f*_ ≡ *γ* for (a)–(d). Except for the variable in (a)–(d), the other parameters for these simulations are shown in Table 2.

In a realistic experiment, the energy relaxation rate *γ* and the anharmonicity *δ* = *ω_g_*_,*e*_ − *ω_e_*_,*f*_ of the qutrit, the decay rate *κ* of the resonator, and the minimum value of tunable coupling strength *g_min_* influence the fidelity of our cc-phase gate. Their effects are shown in Fig. 5 (a)–(d) in which we simulate the fidelity of the gate by varying a single parameter and fixing the other parameters. In Fig. 5 (b), although the fidelity of the cc-phase gate is reduced obviously when the anharmonicity of the qutrit becomes small, it can in principle be improved by taking a smaller coupling strength for the resonance operation.

## Discussion

The number-state-dependent interaction between a superconducting qubit and resonator qudits is an important nonlinear effect which has been used to construct the quantum entangled states and quantum logic gates on resonator qudits in the previous works^53^,^54^,^55^,^66^. This effect is a useful second-order coupling between the qubit and the resonator in the dispersive regime, which indicates a slow operation of the state-dependent selective rotation on the qubit with a drive field. In contrary, our gates are achieved by using the quantum resonance operation only, which is not the high-order coupling item of the qubit and the resonator, and has been realized for generating the Fock states in a superconducting resonator with a high fidelity^44^. All-resonance-based quantum operations make our universal quantum gates on microwave-photon resonators have a shorter operation time, compared with those in previous works^55^. Moreover, our gates have a higher fidelity than those in the latter if we take the decoherence of the qubit and the decay of the resonators into account. Although there are nine steps in constructing our cc-phase gate on three resonators, compared with the three steps in constructing our c-phase gate, the total period of the resonance operations in our cc-phase gate is not much longer than the one in our c-phase gate.

In our simulations, the quantum errors from the preparation of the initial states of Eqs. (2) and (10) are not considered. Single-qubit operations on a qubit^40^ have been realized with the error smaller than 10^−4^ and it can be depressed to much small^67^. That is to say, the error from single-qubit operations has only a negligible influence on the results of the fidelities of our fast universal quantum gates. There are several methods which can help us to turn on and off the resonance interaction between a superconducting qutrit and a resonator, such as tuning the frequency of the qutrit, tuning the frequency of the resonator, or tuning their coupling strength. In experiment, a tunable coupling superconducting device has been realized^59^,^68^. The coupling strength between a phase qubit and a lumped element resonator^68^ can be tuned from 0 MHz to 100 MHz. The coupling strength between a charge qubit and a resonator^59^ can be tuned from 200 KHz to 43 MHz. Tuning the frequency of a high quality resonator has also been realized^69^. The frequency of a 1D superconducting resonator with the quality of 10^4^ can be tuned with a range of 740 MHz. The frequency of a transmon qubit^70^ can be tuned in a range of about 2.5 GHz. In the system composed of several resonators coupled to a superconducting qutrit, by tuning the frequency of the qutrit only to complete the resonance operation between the qutrit and the resonators with a high fidelity, one should take small coupling strengths between them, which leads to a long-time operation^61^. By using the tunable resonator or tunable coupling strength only to turn on and off the interaction, the fast high-fidelity resonance operation requires a much larger tunable range. Here, we tune the frequency of the qutrit and the coupling strengths between the qutrit and each resonator to turn on and off their resonance interactions to achieve our fast universal gates. The coupling strengths are chosen much smaller than the anharmonicity of the transmon qutrit, which helps us to treat the qutrit as a qubit^70^ during the resonance operations without considering the effect from the third excited energy level of the qutrit. To implement our gates in experiment with a high fidelity, one should also apply a magnetic flux with fast tunability. On one hand, it can tune the frequency of the qutrit instantaneously to get the high-fidelity resonance operation^71^. On the other hand, it can help us to get a fast tunable coupling strength between the qutrit and the resonator^59^,^68^.

In summary, we have proposed two schemes for the construction of universal quantum gates on resonator qubits in the processor composed of multiple high-quality microwave-photon resonators coupled to a transmon qutrit, including the c-phase and cc-phase gates. Different from the ones in the previous works based on the dispersive coupling effect of the number-state-dependent interaction between a superconducting qubit and the resonator qubits^55^, our gates are achieved by all-resonance quantum operations and they have the advantages of higher fidelities and shorter operation times. With the optimal feasible parameters, our numerical simulations show that the fidelity of our c-phase gate approaches 99.57% within the operation time of 38.1 ns and that of our cc-phase gate is 99.25% within 73.3 ns, not resorting to drive fields.

## Methods

### Quantum resonance operation

Quantum resonance operation is the key element for the construction of our all-resonance-based universal quantum gates on microwave-photon resonators. In a system composed of a two-energy-level qubit coupled to a cavity, the Hamiltonian of the system is (in the interaction picture)^72^
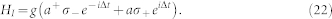
Here Δ = *ω_c_* − *ω_q_* and *ω_c_* is the frequency of the cavity. The Hamiltonian *H_I_* describes the state transfer between the qubit and the cavity. In the system, the unitary time-evolution operation is given by 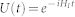, which can be expanded at the exact resonances between the qubit and the cavity (Δ = 0) as^72^
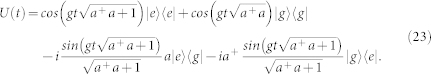


**Figure 6:**
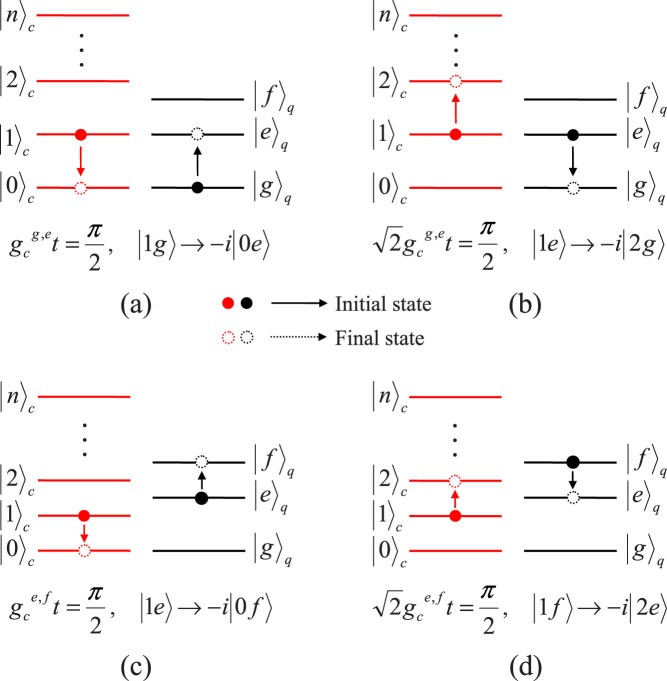
Schematic diagram for the resonance processes between a single-model cavity field and a qutrit. |*n*〉*_c_* is the Fock state of the cavity. *t* is the operation time of the resonance processing.

In our work, the resonance interactions take place between a three-energy-level qutrit and a single-model cavity field. To keep the resonance operation between the cavity and the qutrit with the wanted transition |*g*〉 ↔ |*e*〉 or |*e*〉 ↔ |*f*〉, one should take a small coupling strength between the qutrit and the cavity, compared with the anharmonicity of the qutrit, to avoid the off-resonance interaction between the cavity and the qutrit with the unwanted transition. The details of the state evolution of the system composed of a qutrit and a cavity are described in Fig. 6 (in which we give all the resonance processes used in this work only). In the quantum resonance operation between the cavity and the qutrit in the transmission between the energy levels |*g*〉 and |*e*〉, the evolution |1*g*〉 → −*i*|0*e*〉 (|0*e*〉 → −*i*|1*g*〉) is completed with 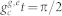, shown in Fig. 6 (a). With 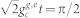, the evolution |1*e*〉 → −*i*|2*g*〉 (|2*g*〉 → −*i*|1*e*〉) can be achieved, shown in Fig. 6 (b). In the quantum resonance operation between the cavity and the qutrit in the transmission between the energy levels |*e*〉 and |*f*〉, the evolution |1*e*〉 → −*i*|0*f*〉 (|0*f*〉 → −*i*|1*e*〉) is completed with 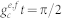, shown in Fig. 6 (c). With 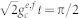, the evolution |1*f*〉 → −*i*|2*e*〉 (|2*e*〉 → −*i*|1*f*〉) can be achieved, shown in Fig. 6 (d).

## Author Contributions

M.H. and M.J. completed the calculation and prepared the figures. M.H. and F.G. wrote the main manuscript text. F.G. supervised the whole project. All authors reviewed the manuscript.

## Figures and Tables

**Table 1:** The steps for constructing the SWAP gate on *r_a_* and *r_b_* with all-resonance operations

Step	Coupling	Time	Transition
i)	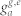	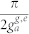	|0〉*_a_*_,_ |1〉*_a_* → |*g*〉*_q_*, |*e*〉*_q_*
ii)	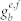	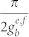	|0〉*_b_*_,_ |1〉*_b_* → |*e*〉*_q_*, |*f*〉*_q_*
iii)	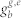	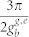	|0〉*_b_*_,_ |1〉*_b_* → |*g*〉*_q_*, |*e*〉*_q_*
iv)	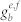	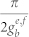	|0〉*_b_*_,_ |1〉*_b_* → |*e*〉*_q_*, |*f*〉*_q_*
v)	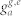	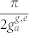	|0〉*_a_*_,_ |1〉*_a_* → |*g*〉*_q_*, |*e*〉*_q_*

**Table 2:** The parameters for constructing the cc-phase gate on *r_a_*_,_ *r_b_*_,_ and *r_c_*

Step	*ω_g_*_,*e*_/(2*π*) (GHZ)	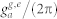 (MHZ)	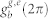 (MHZ)	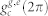 (MHZ)
i)	5.5	45	0.5	0.5
ii)	7.8	0.5	28	0.5
iii)	5.5	27	0.5	0.5
iv)	6.3	24	0.5	0.5
v)	8.8	0.5	0.5	20
vi)	6.3	29	0.5	0.5
vii)	5.5	27	0.5	0.5
viii)	7.8	0.5	28	0.5
ix)	5.5	45	0.5	0.5
